# CXCR3 antagonist VUF10085 binds to an intrahelical site distinct from that of the broad spectrum antagonist TAK–779

**DOI:** 10.1111/bph.13027

**Published:** 2015-02-10

**Authors:** Belinda Nedjai, Jonathan M Viney, Hubert Li, Caroline Hull, Caroline A Anderson, Tomoki Horie, Richard Horuk, Nagarajan Vaidehi, James E Pease

**Affiliations:** 1 Leukocyte Biology Section, NHLI Division, Faculty of Medicine, Imperial CollegeLondon, UK; 2 Department of Immunology, Beckman Research Institute of the City of HopeDuarte, CA, USA; 3 Tokyo Medical and Dental UniversityTokyo, Japan; 4 Department of Pharmacology, University of CaliforniaDavis, CA, USA

## Abstract

**Background and Purpose:**

The chemokine receptor CXCR3 is implicated in a variety of clinically important diseases, notably rheumatoid arthritis and atherosclerosis. Consequently, antagonists of CXCR3 are of therapeutic interest. In this study, we set out to characterize binding sites of the specific low MW CXCR3 antagonist VUF10085 and the broad spectrum antagonist TAK-779 which blocks CXCR3 along with CCR2 and CCR5.

**Experimental Approach:**

Molecular modelling of CXCR3, followed by virtual ligand docking, highlighted several CXCR3 residues likely to contact either antagonist, notably a conserved aspartate in helix 2 (Asp-112^2:63^), which was postulated to interact with the quaternary nitrogen of TAK-779. Validation of modelling was carried out by site-directed mutagenesis of CXCR3, followed by assays of cell surface expression, ligand binding and receptor activation.

**Key Results:**

Mutation of Asn-132^3.33^, Phe-207 and Tyr-271^6.51^ within CXCR3 severely impaired both ligand binding and chemotactic responses, suggesting that these residues are critical for maintenance of a functional CXCR3 conformation. Contrary to our hypothesis, mutation of Asp-112^2:63^ had no observable effects on TAK-779 activity, but clearly decreased the antagonist potency of VUF 10085. Likewise, mutations of Phe-131^3.32^, Ile-279^6.59^ and Tyr-308^7.43^ were well tolerated and were critical for the antagonist activity of VUF 10085 but not for that of TAK-779.

**Conclusions and Implications:**

This more detailed definition of a binding pocket within CXCR3 for low MW antagonists should facilitate the rational design of newer CXCR3 antagonists, with obvious clinical potential.

## Tables of Links

**Table:** 

TARGETS
**GPCRs**
CCR1
CCR2
CCR3
CCR5
CXCR3
CXCR4

**Table:** 

LIGANDS
CXCL9 (Mig)
CXCL10 (IP-10)
IT1t
UCB 35625

These Tables list key protein targets and ligands in this article which are hyperlinked to corresponding entries in http://www.guidetopharmacology.org, the common portal for data from the IUPHAR/BPS Guide to PHARMACOLOGY (Pawson *et al*., 2014) and are permanently archived in the Concise Guide to PHARMACOLOGY 2013/14 (*^a,b,c^*Alexander *et al*., 2013).

## Introduction

The recruitment of leukocytes from the circulation to the tissues is coordinated to a large extent by chemokines, a family of around 40 proteins in humans (Zlotnik and Yoshie, 2012). Chemokines bind to specific chemokine receptors located on the leukocyte surface and drive chemotaxis, the directional migration of the cell along the chemokine concentration gradient. Ordinarily, this is a desirable process, populating tissues with leukocytes to provide protection against invading microorganisms. However, in several clinically important diseases, the inappropriate or excessive production of chemokines is associated with increased leukocyte recruitment and tissue damage. Consequently, the notion of blocking chemokine receptors with small molecule antagonists has gained momentum in the field of medicinal chemistry, with several candidate molecules being developed and entering clinical trials (see Pease and Horuk, 2012).

The CXC chemokine receptor CXCR3 is expressed on the surface of a variety of leukocytes, most notably T-cells and, like all other chemokine receptors, is a 7TM (7 transmembrane helix) receptor, binding the chemokines CXCL9 (Mig), CXCL10 (IP-10) and CXCL11 (I-TAC) with affinities in the low nanomolar range (Cole *et al*., 1998). CXCL9, CXCL10 and CXCL11 are all induced by IFN-γ and therefore thought to promote Th1 immune responses (Luster and Ravetch, 1987; Farber, 1990; Cole *et al*., 1998), and play key roles in the inflammatory responses seen in rheumatoid arthritis (Ruschpler *et al*., 2003), atherosclerosis (Mach *et al*., 1999), psoriasis (Flier *et al*., 2001) and transplant rejection (Hancock *et al*., 2001). Accordingly, CXCR3 has attracted much attention as a therapeutic target for the treatment of these diseases, with several low MW antagonists of CXCR3 described by ourselves and others (Johnson *et al*., 2007; Hayes *et al*., 2008; Li *et al*., 2008; Verzijl *et al*., 2008; Liu *et al*., 2009; Chan *et al*., 2011; Wijtmans *et al*., 2011; Jenh *et al*., 2012) . Although several studies of CXCR3 antagonists have shown their efficacy in *in vivo* models of disease, notably atherosclerosis (van Wanrooij *et al*., 2008) and transplant rejection (Jenh *et al*., 2012), only one such antagonist has entered clinical trials in man, the compound AMG-487, originally identified by scientists at Chemocentryx and subsequently licensed to Amgen. Preclinical data showed AMG-487 to have excellent potency and efficacy in the inhibition of immune cell migration and efficacy in a bleomycin-induced model of lung inflammation in mice (Johnson *et al*., 2007). However, in phase II clinical trials for the treatment of psoriasis, AMG-487 failed to demonstrate efficacy leading to termination of the trial (Ribeiro and Horuk, 2005). This has led to the hypothesis that in certain inflammatory disorders where several chemokines are induced, it may be necessary to block more than one receptor to achieve efficacy (Pease and Horuk, 2010).

**Figure 1:**
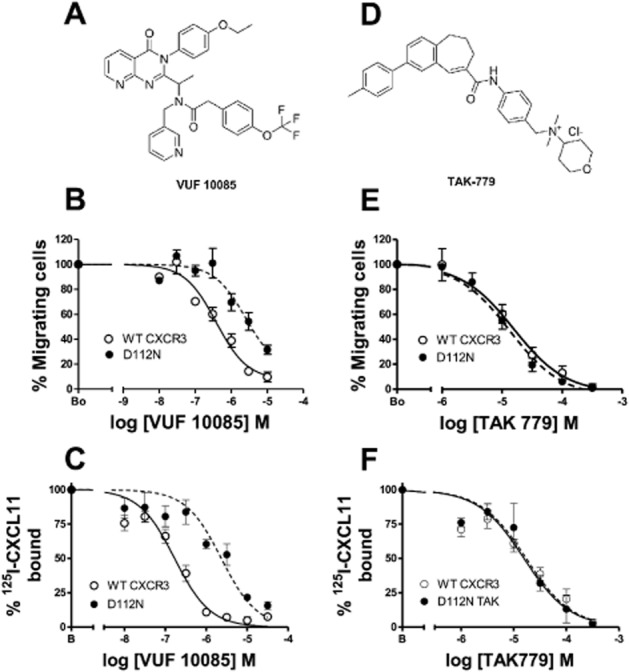
Mutation of Asp-112 of CXCR3 reduces the antagonist activity of VUF 10085 but has no effect upon TAK-779 activity. (A) The structures of VUF 10085 and (D) of TAK-779. B and E: the migration of WT CXCR3 transfectants and D112N-CXCR3 expressing transfectants in response to a fixed concentration of 10 nM CXCL11 and increasing concentrations of VUF 10085 (B) or TAK-779 (E). IC_50_ values for inhibition by VUF 10085 were 380 nM (WT) and 2.52 μM (D112N); IC_50_ values for inhibition by TAK-779 were 1.56 μM (WT) and 1.34 μM (D112N); (C and F) the displacement of ^125^I-CXCL11 from WT CXCR3 transfectants and D112N-CXCR3 expressing transfectants in response to increasing concentrations of VUF 10085 (C) or TAK-779 (F). IC_50_ values for inhibition by VUF 10085 were 169 nM (WT) and 2.34 μM (D112N); IC_50_ values for inhibition by TAK-779 were 15.6 μM (WT) and 17.2 μM (D112N). *n* = 3 separate experiments.

The low MW compound TAK-779 (Fig 1D) was originally developed by Takeda Pharmaceutical Company as a prototypic low MW antagonist of CCR5, and shown to be an inhibitor of HIV-1 entry *in vitro* (Baba *et al*., 1999), binding to an intrahelical site within the receptor (Dragic *et al*., 2000; Charo and Ransohoff, 2006; Hall *et al*., 2009). As CCR5 also shares 74% identity with the receptor CCR2, it is perhaps unsurprising that the molecule also has excellent potency and efficacy at the latter receptor and, via a programme of *ab initio* receptor modelling and site-directed mutagenesis, we have been able to compare the binding sites of this molecule in both CCR2 and CCR5 (Hall *et al*., 2009). What makes TAK-779 unique among current prototypic antagonists, is that it also has reasonable potency and efficacy at a relatively unrelated chemokine receptor of a different class, namely CXCR3 (Gao *et al*., 2003; Verzijl *et al*., 2008). Several *in vivo* studies have demonstrated the efficacy of TAK-779 in Th1 dominated diseases such as collagen-induced arthritis (Yang *et al*., 2002) colitis (Tokuyama *et al*., 2005), allograft rejection (Akashi *et al*., 2005) and ischaemia/reperfusion injury (Akahori *et al*., 2006), which is likely to be related to its broad spectrum activity in blocking CCR2, CCR5 and CXCR3 in both humans and rodents. More detailed knowledge of how a single compound interacts with three distinct chemokine receptors should facilitate the discovery of similar broad spectrum antagonists with therapeutic potential.

We describe here a programme of research in which *ab initio* modelling of CXCR3 coupled with site-directed mutagenesis and assays of receptor activation were used to characterize the binding sites of two known CXCR3 antagonists, the 3*H*-pyrido[2,3-*d*]pyrimidin-4-one derivative *N*-1*R*-[3-(4-ethoxy-phenyl)-4-oxo-3,4-dihydro-pyrido[2,3-*d*]pyrimidin-2-yl]-ethyl-*N*-pyridin-3-ylmethyl-2-(4-trifluoromethoxy-phenyl)-acetamide (VUF10085/AMG-487) and the broad spectrum quaternary ammonium anilide *N*,*N*-dimethyl-*N*-[4-[[[2-(4-methylphenyl)-6,7-dihydro-5*H*-benzocyclohepten-8-yl]-carbonyl]amino]benzyl] tetrahydro-2*H*-pyran-4-aminium chloride (TAK-779).

## Methods

### Generation of receptor mutants and their transient expression in the murine pre-B cell line L1.2

A previously described pcDNA3 plasmid containing human wild type (WT) CXCR3 cDNA with an HA epitope tag encoded at the N terminus (Meiser *et al*., 2008) was used as a template for the generation of point mutants by PCR using the QuikChange II site-directed mutagenesis kit (Stratagene, Amsterdam, the Netherlands). All constructs were verified by DNA sequencing (Eurofins MWG Operon, Ebersberg, Germany) before use. L1.2 cells were transiently transfected by electroporation with 1 μg of vector DNA/10^6^ cells at 330V, 975μF and incubated overnight in medium supplemented with 10 mM of sodium butyrate to enhance gene expression.

### Flow cytometry

Cell surface expression of CXCR3 was assessed by flow cytometry after staining with an anti-HA antibody and FITC-conjugated secondary antibody as described previously (Vaidehi *et al*., 2009). Expression was analysed using a FACSCalibur flow cytometer (Becton Dickinson, Mountain View, CA, USA). Data are presented as a percentage of the amount of WT CXCR3 expressed in control transfectants.

### Chemotaxis assay

Assays of chemotactic responsiveness were carried out as previously described (Vaidehi *et al*., 2009) using 96-well ChemoTx® plates with 5 μm pores (Neuroprobe, Gaithersburg, MD, USA). Migrating cells were detected by the use of CellTiter Glo® Dye (Promega, Southampton, UK) and resulting luminescence measured using a TopCount scintillation counter (PerkinElmer, Waltham, MA, USA). Basal migration of cells to buffer alone was subtracted from the resulting data, with individual results expressed as a percentage of the total cells applied to the filter. In all experiments, each data point was assayed in duplicate. In every experiment, cells transiently expressing WT CXCR3 were used as a positive control.

### Radiolabelled chemokine binding studies

Whole-cell binding assays on transiently transfected L1.2 cells were performed as described previously (Vaidehi *et al*., 2009) using 0.1 nM ^125^I-CXCL11 (Perkin Elmer) and increasing concentrations of unlabeled CXCL11 or antagonist. Cell-associated radioactivity was counted in a Canberra Packard Cobra 5010 gamma counter (Canberra Packard, Pangebourne, UK). Curve fitting and subsequent data analysis was carried out using the program PRISM (GraphPad Software, Inc., San Diego, CA, USA) and IC_50_ values were obtained by non-linear regression analysis. In all experiments, each data point was assayed in duplicate, with each individual experiment repeated three times. Background binding levels obtained in the presence of a 1000–3000 molar excess of unlabelled chemokine were subtracted from each data point and data are presented as the percentage of counts obtained in the absence of competing ligand. *K*_D_ values and the number of binding sites per cell were calculated from homologous binding curves prepared in Graph Pad Prism (La Jolla, CA, USA) as previously described (Nedjai *et al*., 2011).

### Modelling the CXCR3 interaction with VUF 10085

The three-dimensional model of the seven helical TM bundle of human CXCR3 was predicted using the *ab initio* method MembStruk (Vaidehi *et al*., 2002; Hall *et al*., 2009). The extra and intracellular loops were added using the method, Modeller. VUF 10085 was built using the LigPrep module from the Schrodinger Glide suite (Schrodinger Inc.). Multiple ligand conformations were generated for the compound and docked using Glide XP (Schrodinger Inc., Portland, OR, USA). Subsequently, a short energy minimization was performed on each docked pose and the binding energy of this optimized pose was calculated. The binding energy was calculated as BE (binding energy) = PE (ligand in fixed protein) − PE (ligand in solvation); where BE is the binding energy and PE is the potential energy. The compound poses were then sorted by binding energy and the top 20 conformations inspected visually to maximize the interactions with residues that are known to interact with ligands in chemokine receptors (Vaidehi *et al*., 2009). During the course of this work, the crystal structure of CXCR4 bound to a low MW antagonist was published (Wu *et al*., 2010). Therefore, we also generated a homology model of CXCR3 based on CXCR4 crystal structure as template (pdb ID:3ODU) using the program MODELLER (http://salilab.org/modeller/9v7/manual/node8.html). We selected the top 100 models from MODELLER and clustered these models by their root mean squared deviation in coordinates. The 100 models clustered into five clusters and the best energy structure from the cluster was chosen for docking. We then docked the VUF 10085 antagonist to this model using GOLD (http://www.ccdc.cam.ac.uk/SupportandResources/Support/pages/SupportSolution.aspx?supportsolutionid=110) flexible side chain docking to allow for protein flexibility. The side chains of the residues Y60, W109, D112, F131, F135, H202, Y271 and Y308 were treated as flexible using the built in rotamer library. A distance constraint was placed between D112 and the pyridine nitrogen of the VUF 10085 compound. The best docked pose was selected based on the experimental data in this paper. The final model was used in generating Figure 7A–C.

### Data analysis

Data are expressed as the mean ± SEM of the number of experiments indicated in the Figure legends.

### Materials

Reagents were purchased from Invitrogen (Paisley, UK), unless stated otherwise. Recombinant human CXCL10 and CXCL11 were purchased from PeproTech EC, Ltd. (London, UK). The monoclonal mouse anti-haemagglutinin (HA) anti-HA.11 antibody was from Covance (Berkeley, CA, USA) and its corresponding IgG1 isotype control antibody from Sigma-Aldrich (Poole, UK). The anti-CXCR3 mAb (Clone 49801) was from R&D Systems (Abingdon, UK). The murine pre-B cell line L1.2 was maintained as described previously (Vaidehi *et al*., 2009). TAK-779 was obtained from the Programme EVA Centre for AIDS Reagents, NIBSC, UK, supported by the EC FP6/7 Europrise Network of Excellence, AVIP and NGIN consortia and the Bill and Melinda Gates GHRC-CAVD Project and was donated by Dr R. Gallo, University of Maryland School of Medicine. The synthesis of VUF 10085 has previously been described (Flier *et al*., 2001; Storelli *et al*., 2007).

### Nomenclature

Ballesteros–Weinstein numbering (Ballesteros and Weinstein, 1995) is used in superscript throughout the text to denote the positioning of residues within the TM helices. In this nomenclature, a single most conserved residue among the class A GPCRs is designated x.50, where x is the TM helix number; all other residues on that helix are numbered relative to this conserved position.

## Results

We have previously described the potency and efficacy of the specific low MW CXCR3 antagonist VUF10085 (Figure 1A and the broad spectrum antagonist TAK-779 (Figure 1D) at human CXCR3, with VUF 10085 the more potent of the two compounds in a variety of assays (Verzijl *et al*., 2008). In this study, we set out to elucidate the binding site of both compounds within the CXCR3 structure. Ourselves and others have previously shown that small molecule chemokine receptor antagonists with a quaternary nitrogen, as typified by TAK-779, often form a salt bridge with a highly conserved glutamate in TM helix 7 (Glu^7.39^) when docking to the receptor (Dragic *et al*., 2000; de Mendonça *et al*., 2005; Vaidehi *et al*., 2006; Wise *et al*., 2007; Hall *et al*., 2009; Pease and Horuk, 2010). As CXCR3 is highly unusual among the chemokine receptor family in not possessing a glutamate at this position, we turned our attention to an aspartate in helix two, Asp-112^2:63^. We hypothesized that this would be a likely partner for bonding with the positively charged quaternary nitrogen of TAK-779 but be dispensable (i.e., not critical) for the binding of VUF 10085. We have previously studied the role of Asp-112^2:63^ in CXCR3 activation by chemokine and found that the residue was critical for activation by the chemokine CXCL10, but not by CXCL11 (Nedjai *et al*., 2011). Consequently, all assays employed CXCL11 as an agonist. In disagreement with our original hypothesis, mutation of Asp-112^2:63^ to asparagine (D112N^2:63^) had considerable effects upon the ability of VUF 10085 to inhibit CXCL11-induced cell migration (Figure 1B), with a sixfold increase in the relative IC_50_ values (WT = 380 nM, D112N = 2.52 μM). In contrast, little difference in the IC_50_ values for TAK-779 inhibition was observed (Figure 1E, WT = 1.56 μM and D112N = 1.34 μM). Similarly, mutation of D112 decreased the ability of VUF 10085 to inhibit CXCL11 binding (Figure 1C), with a 13-fold increase in the relative IC_50_ values (WT = 169 nM, D112N =2.34 μM. In contrast, IC_50_ values for TAK-779 inhibition were very similar (WT = 15.6 μM and D112N = 17.2 μM, Figure 1F). Thus, we conclude that an acidic residue in TM helix II, Asp-112^2:63^, acts as an anchor point for VUF 10085 but not for TAK-779.

**Figure 2:**
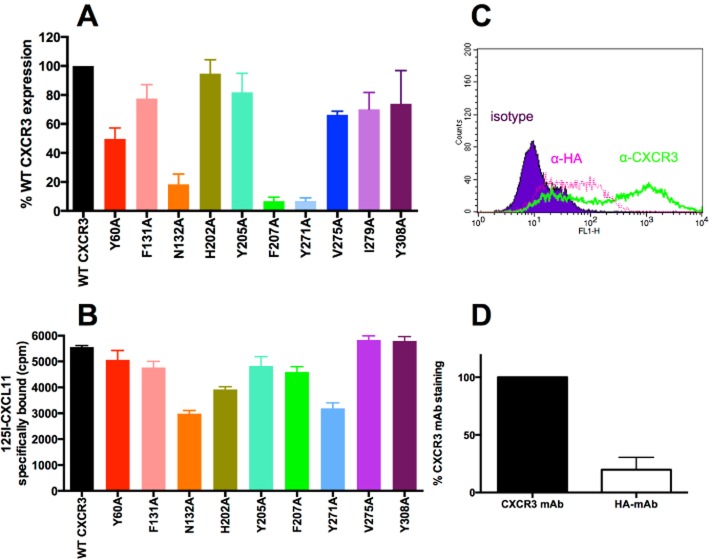
Relative cell surface levels of WT CXCR3 and mutant CXCR3 constructs. Panel A shows cell surface expression of CXCR3 mutants relative to that of WT CXCR3 as assessed by flow cytometry using an antibody against an N-terminally incorporated HA epitope. *n* = 3–9 separate experiments in each case. Panel B shows the relative levels of ^125^I-CXCL11 bound by the same panel of transfectants, *n* = 3 separate experiments. Panels C and D show comparative staining of WT CXCR3 with the anti-HA mAb and an anti-CXCR3 mAb, *n* = 3.

We subsequently explored further the binding site of the two CXCR3 antagonists by computational modelling using *ab initio* derived structures of CXCR3 (Vaidehi *et al*., 2009) docked with either VUF 10085 or TAK-779. The preliminary models thus obtained suggested an additional 10 residues likely to interact with the antagonists (Table 1), which were concentrated in five TM helices and the second extracellular loop (ECL2). Plasmids were generated in which the codon encoding each of these residues was singularly mutated to one encoding alanine and following transient transfection of cells, the effects of mutation upon CXCR3 cell surface expression and ligand binding were examined. As in previous studies, a conformationally insensitive N-terminal HA epitope was introduced into the constructs to aid detection at the cell surface by flow cytometry. Expression of the CXCR3 mutants was on the whole robust, with only mutation of Tyr-60^1.39^, Asn-132^3.33^, Phe-207 and Tyr-271^6.51^ decreasing detection of CXCR3 at the cell surface (Figure 2A). Notably, the F207A and Y271A^6.51^ mutants were expressed at levels barely above those of the isotype control antibody suggesting that they did not traffic to the cell surface. However, when the ability of the same panel of transfectants to bind ^125^I-CXCL11 was examined, an imperfect correlation between detection of the HA epitope and CXCL11 binding was observed, with the Y60A^1.39^ and F207A mutants binding levels of ^125^I-CXCL11 not dissimilar to those binding to WT CXCR3. This suggests that the accessibility of the anti-HA antibody to the N-terminal HA epitope of our CXCR3 construct may be restricted by the native conformational of CXCR3. This postulate was supported when the relative abilities of the anti-HA antibody and an anti-CXCR3 mAb to recognize WT CXCR3 were directly compared, with an approximate 75% reduction in CXCR3 detection levels observed between the two antibodies Figure 2C and D).

**Table 1:** Expression, chemotaxis and binding properties of CXCR3 mutants

Construct	% of WT CXCR3 surface expression		% of WT chemotaxis to 30 nM CXCL11		*K*_D_ CXCL11 binding (nM)		Number of receptors per cell	
	Mean ± SEM	*n*	Mean ± SEM	*n*	Mean ± SEM	*n*	Mean ± SEM	*n*
WT CXCR3	100 ± 1.6	9	100 ± 4.32	3	7.7 ± 1.4	3	51 843 ± 8678	3
Y60A	49.8 ± 7.5	9	55.4 ± 16.4	3	1.8 ± 0.4	3	10 500 ± 3130	3
F131A	77.6 ± 9.5	9	53.7 ± 5.6	3	1.1 ± 0.2	3	7578 ± 1312	3
N132A	18.4 ± 7.1	8	0.7 ± 0.5	3	ND		ND	
H202A	95.3 ± 9.6	9	66.0 ± 34.8	3	2.9 ± 1.3	3	9292 ± 5332	3
Y205A	82.0 ± 13	8	35.5 ± 21.5	3	10.4 ± 1.2	3	13 124 ± 1398	3
F207A	6.8 ± 2.8	3	0.8 ± 0.8	3	ND		ND	
Y271A	6.8 ± 2.3	3	1.0 ± 0.6	3	ND		ND	
V275A	66.3 ± 2.5	9	52.9 ± 8.2	3	7.4 ± 0.9	3	54 219 ± 2561	3
I279A	70.2 ± 11.6	9	51.3 ± 6.2	3	ND		ND	

Surface expression and chemotaxis are expressed as the percentage of the mean of the values obtained for transfectants expressing WT CXCR3. *n*, numbers of experiments. ND indicates not determined.

**Figure 3:**
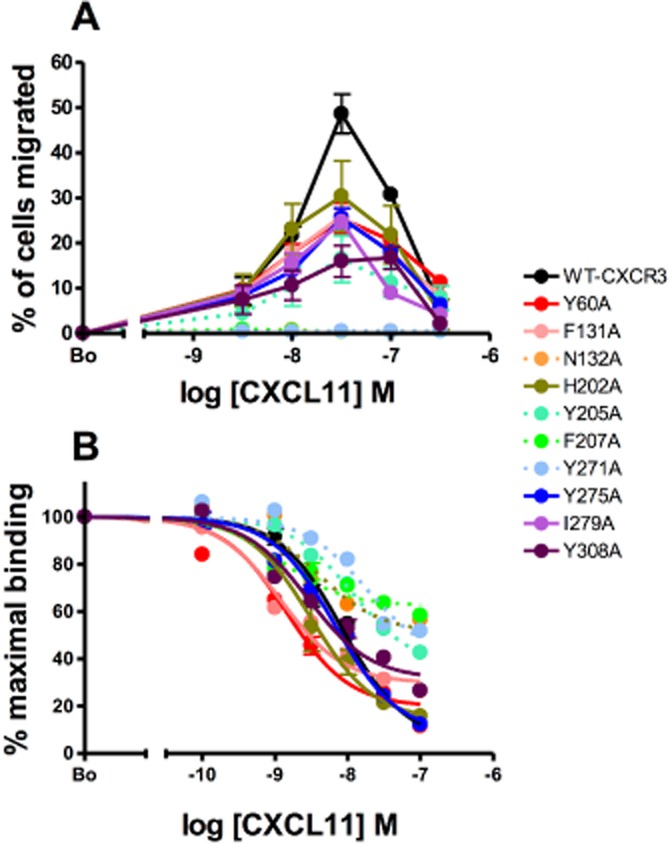
Effects of site-directed mutagenesis upon CXCR3 function. Panel A shows the chemotactic responses of transfectants expressing either WT CXCR3 or a panel of point mutants. Panel B shows the ability of the same panel of mutants to bind ^125^I-CXCL11 as determined by competition binding assays employing unlabelled CXCL11. *n* = 3 separate experiments in both panels.

We subsequently assessed the panel of CXCR3 mutants for their ability to bind and signal in response to CXCL11, using chemotaxis assays and competitive binding assays. WT CXCR3 behaved as previously reported (Xanthou *et al*., 2003) with a bell-shaped chemotaxis response reaching an optimum at a concentration of 30 nM CXCL11 (Figure 3A). Of the panel of CXCR3 constructs assessed, three mutations were seen to severely affect the chemotactic response, namely the N132A^3.33^, F207A and Y271A^6.51^ constructs (Table 1). The remaining constructs all exhibited a bell-shaped response of similar potency, albeit with reduced efficacy. In competitive binding assays, WT CXCR3 bound CXCL11with a *K*_D_ of 7.7 nM (Figure 3B, Table 1) with several mutations appearing to decrease the apparent *K*_D_. This was explained when non-linear regression analysis of the CXCL11 data was carried out to determine the number of receptors per cell. Decreases in the apparent *K*_D_ of mutant receptors were found to correlate with reduced receptor expression (Table 1). In keeping with their lack of chemotactic activity, the maximum concentration of CXCL11 was unable to displace 50% of the ^125^I-CXCL11 at the N132A^3.33^, F207A and Y271A^6.51^ mutants (Figure 3B), suggesting that Asn-132^3.33^, Phe-207 and Tyr-271^6.51^ are required for the integrity of a functional CXCR3 conformation.

**Figure 4:**
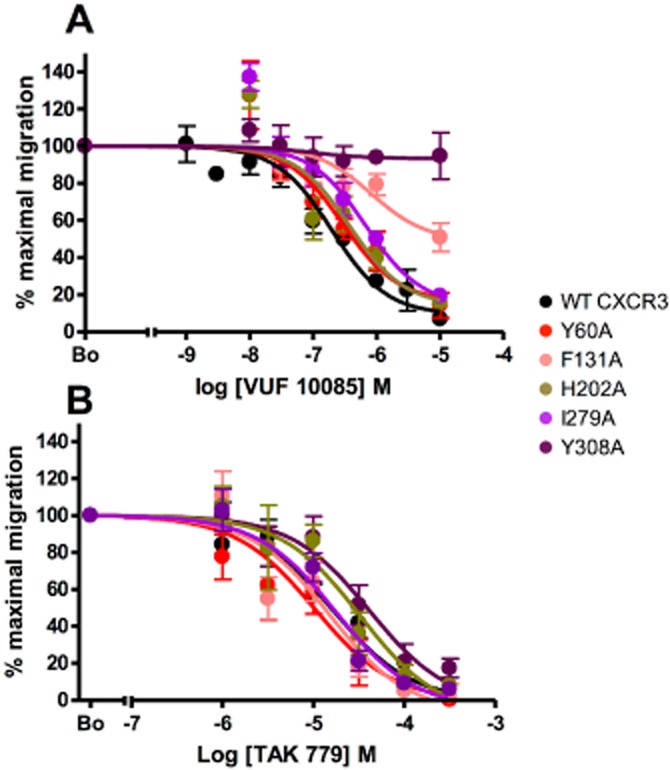
Mutation of intrahelical residues inhibits the antagonist activity of VUF 10085 but not TAK-779 in chemotaxis assays. (A and B) The relative abilities of increasing concentrations of VUF 10085 (A) and TAK-779 (B) to inhibit the migration of CXCR3 transfectants to 30 nM CXCL11. *n* = 3 separate experiments in both panels.

The functional CXCR3 constructs were subsequently assessed for their ability to be antagonized by either VUF 10085 or TAK-779 in chemotaxis assays, using the optimal 30 nM concentration of CXCL11 to drive cell migration (Figure 4A and B). In these assays, a construct showing a loss of sensitivity to either compound is interpreted as highlighting a CXCR3 residue contacting the antagonist. In the assessment of VUF 10085, three mutant constructs decreased the ability of VUF 10085 to inhibit chemotactic responses to CXCL11 (Figure 4A, Table 2). Notably, the Tyr-308A^7.43^ and Phe-131A^3.32^ mutations rendered VUF 10085 impotent, with calculation of an IC_50_ value impossible. Similarly, mutation of Ile-279^6.59^ resulted in a threefold increase in the IC_50_ value for VUF 10085. Mutation of Tyr-60^1.39^ and His-202 increased the IC_50_ values for VUF 10085, but to a lesser degree. In contrast, the ability of TAK-779 to inhibit chemotaxis was hardly changed by mutation of the same five CXCR3 residues (Figure 4B, Table 2), with the H202A and Y308A^7.43^ mutations apparently increasing the IC_50_ values twofold when compared with WT CXCR3.

**Table 2:** Inhibition of chemotaxis and ligand binding by VUF 10085 and TAK-779 at WT and mutant CXCR3 constructs

	VUF 10085		TAK-779	
Construct	IC_50_ chemotaxis (mean ± SEM; μM)	IC_50_ ligand binding (mean ± SEM; μM)	IC_50_ chemotaxis (mean ± SEM; μM)	IC_50_ ligand binding (mean ± SEM; μM)
WT CXCR3	0.198 ± 0.035	0.241 ± 0.027	15.8 ± 3.10	31.3 ± 6.25
Y60A^1.39^	0.292 ± 0.013	0.236 ± 0.054	10.2 ± 4.62	14.0 ± 7.07
F131A^3.32^	ND	0.161 ± 0.040	15.0 ± 6.98	23.3 ± 6.57
H202A (ECL2)	0.373 ± 0.014	0.299 ± 0.120	32.7 ± 14.0	25.3 ± 10.2
I279A^6.59^	0.676 ± 0.028	0.483 ± 0.210	10.5 ± 3.35	39.4 ± 15.0
Y308A^7.43^	ND	ND	41.4 ± 22.6	14.2 ± 3.0

Data are derived from three separate experiments. ND indicates not determined.

**Figure 5:**
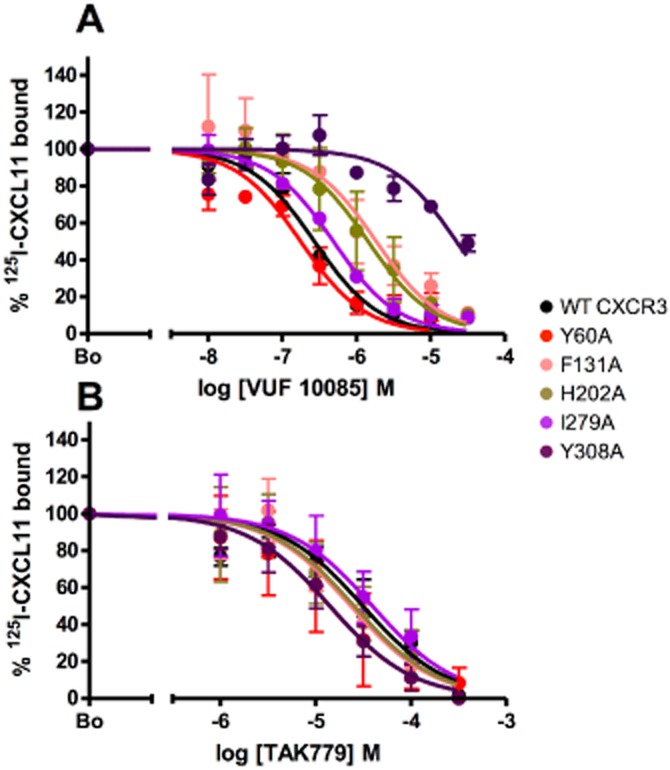
Mutation of several intrahelical residues inhibits the ability of VUF 10085 but not TAK-779 to displace CXCL11 from CXCR3. (A and B) The relative abilities of increasing concentrations of VUF 10085 (A) and TAK-779 (B) to displace 0.1 nM ^125^I-CXCL11 from CXCR3 transfectants. *n* = 3 separate experiments in both panels.

The same panel of CXCR3 mutants was then assessed in ligand binding assays, examining the ability of the antagonists to displace CXCL11 from the receptor (Figure 5A and B). In the assessment of VUF 10085, mutation of Ile-279^6.59^ decreased the potency of VUF 10085 twofold while mutation of Y308^7.43^ decreased the efficacy to such an extent that no IC_50_ value could be calculated (Table 2). In keeping with the chemotaxis data (Figure 4B), none of the five mutations examined appeared to impair the potency or efficacy of TAK-779 in binding assays (Figure 5B, Table 2), with two mutations, Y60A^1.39^ and Y308A^7.43^ resulting in an apparent twofold reduction in the IC_50_ values relative to WT CXCR3.

Taken together with our findings from the chemotaxis assays, we conclude that while VUF 10085 binds within an intrahelical site containing residues Phe-131^3.32^, Ile-279^6.59^ and Tyr-308^7.43^ of CXCR3, the broad spectrum antagonist TAK-779 binds to a distinct site, which does not involve these residues.

**Figure 6:**
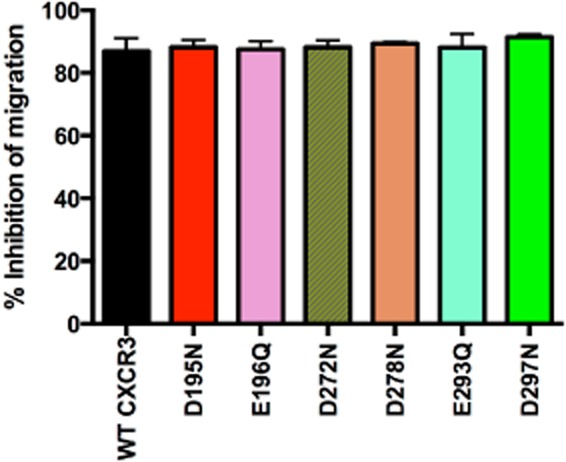
Mutation of charged extracellular residues has no effect upon the ability of TAK-779 to inhibit chemotaxis in response to CXCL11. Migration of a panel of CXCR3 transfectants to 30 nM CXCL11 in the presence of 100 μM TAK-779 was determined. Data are expressed as the percentage inhibition of the individual responses to CXCL11 in the presence of vehicle alone. *n* = 3 separate experiments.

We have previously used an alternative panel of six ‘loss of charge’ CXCR3 mutants to determine the counterion for a negatively charged small molecule CXCR3 agonist (Nedjai *et al*., 2011). Although these acidic residues lay outside our initially molecular modelling of the TAK-779:CXCR3 interaction, we tested the hypothesis that one or more of these acidic residues may contribute to TAK-779 binding. As we previously found, all six mutants were well expressed by L1.2 cells and responded robustly in chemotaxis assays to a fixed concentration of 30 nM CXCL11 (data not shown). Contrary to our hypothesis, the ability of TAK-779 to inhibit this migration was not reduced, suggesting that the TAK-779 binding site within CXCR3 does not involve these residues. (Figure 6).

**Figure 7:**
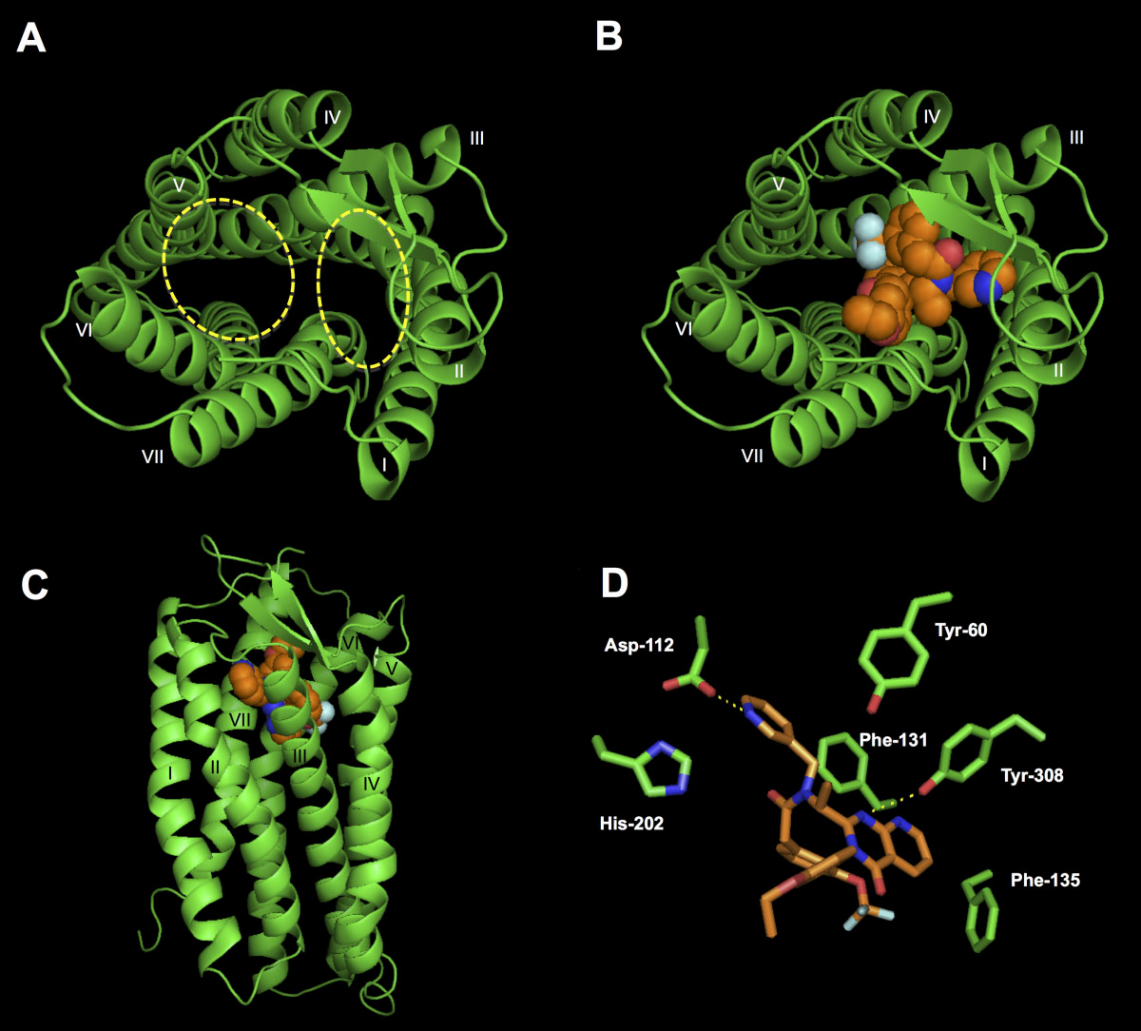
*Ab initio* modelling of CXCR3 and docking of VUF 10085 into the minor binding pocket. (A and B) Top views of a model of human CXCR3 (green) predicted using the software MembStruk. Panel A shoes the major and minor binding pockets, while panel B shows VUF 10085 (orange, space-filled) residing in the minor binding site predicted using Glide XP. Panel C shows a side view of the docked antagonist. Panel D shows key interactions of CXCR3 side chains with the compound. Hydrogen bonds between Asp-112^2.63^ and Tyr-308^7.43^ of CXCR3 with VUF 10085 are denoted by a dashed yellow line. Roman numerals refer to the seven TM helices.

## Discussion and conclusions

We describe here details of the binding site within CXCR3 for the specific antagonist VUF 10085. Preliminary *ab initio* modelling suggested a series of CXCR3 side chains within the TM helices and ECL2, which were likely to interact with VUF 10085 and were necessary for its inhibitory activity. Mutation of these residues coupled with assays of receptor function was used to validate and refine the model. Mutation to alanine of the residues Asn-132^3.33^, Phe-207 (ECL2) and Tyr-271^6.51^ were noted for their deleterious effects upon CXCR3 expression and function, suggesting a role for these side chains in maintaining the correct conformation of the apo-protein. Hence, the possible contribution of these residues to antagonist binding could not be validated experimentally.

In keeping with studies of small molecule antagonists of other chemokine receptors, VUF 10085 binds in the minor pocket of CXCR3 as deduced by molecular modelling and experimental validation (Figure 7A-C). Asp-112^2.63^ is postulated to form a close interaction with the nitrogen of the pyridine ring of VUF 10085 (Figure 7D). An earlier structure–activity relationship (SAR) study by Johnson and colleagues identified the pyridyl group of VUF 10085 as being important for activity in ^125^I-ligand displacement studies (Johnson *et al*., 2007). Tyr-308^7.43^ is predicted to form a close interaction with the nitrogen atom of the quinazolinone ring of the compound. In the SAR study by Johnson and colleagues, this nitrogen atom was present in all of their derivatives and appeared to be important for activity at CXCR3 (Johnson *et al*., 2007). The aromatic sidechain of Phe-131 is believed to stabilize the pyridine ring of VUF 10085 via hydrophobic π–π stacking interactions. Our findings are in general agreement with a recent study published by Scholten and colleagues, which found that mutation of Asp-112^2.63^, F131A^3.32^and Tyr-308^7.43^ impaired the ability of the structurally related compound NBI-74330 to displace CXCL11 binding to CXCR3 (Scholten *et al*., 2013). The overall interaction of CXCR3 with VUF 10085 is not dissimilar to that of another CXC receptor with an antagonist, as revealed by the crystal structure of the low MW antagonist IT1t bound to CXCR4 (Wu *et al*., 2010). In this structure, the analogous residues of CXCR4, Asp-97^2.63^ and Tyr-116^3.32^ form a salt bridge and hydrophobic contact respectively with IT1t. We postulate that Phe-135^3.36^ of CXCR3 contributes to the binding by forming π–π stacking interactions with VUF 10085, although this was untested by our mutagenesis study. Although a potent CXCR3 antagonist *in vitro*, the failure of VUF 10085/AMG-487 to achieve efficacy in the clinic was attributed in part to the production of a metabolite with inhibitory properties at cytochrome P-450 (CYP)-3A, and subsequent non-linear pharmacokinetics following multiple administrations (Henne *et al*., 2012). Occupation of the same minor binding pocket of CXCR3 with a compound with more favourable pharmacokinetics is perhaps worthy of further investigation.

In contrast to our study of VUF 10085, mutation of the panel of intrahelical and ECL2 mutations that form the binding pocket of VUF 10085 had no discernable effect on the ability of TAK-779 to interact with CXCR3, suggesting that its binding site was distinct from that of VUF 10085. This is surprising, as we have previously mapped the binding site of TAK-779 to the minor pockets of both CCR2 and CCR5, where the conserved tyrosine residues at position ^1.39^ and ^3.32^ were critical for antagonist function. However, in this study, when the analogous Tyr-60^1.39^ and Phe-131^3.32^ of CXCR3 were mutated, no impairment of TAK-779 function was observed. This would appear to be against the trend of results from known CC chemokine receptor antagonists, as we previously found that tyrosine residues in the analogous ^1.39^ and ^3.32^ positions of the receptors CCR1 and CCR3 were critical for the function of the bi-specific antagonist UCB 35625 (de Mendonça *et al*., 2005; Wise *et al*., 2007). Thus we have concluded that TAK-779 binds outside the minor pocket of CXCR3.

It should be noted that the possibility for a type II error in our work exists, namely that due to insufficient experimentation, we have failed to show that some of the residues in CXCR3 do form a TAK-779 binding site. For example, these could include the residues N132^3.32^, Y205 and F207 (ECL2) and Y271^6.50^, which were devoid of signalling and therefore could not be subjected to the loss of sensitivity tests to TAK-779 that other mutants were subjected to. The use of additional assays of CXCR3 activation may have been informative in this instance, such as GTPγS binding assays (Smit *et al*., 2003). It is also possible that some of the other residues such as Y308^7.43^, which showed a trend towards decreased sensitivity to TAK-779 in chemotaxis assays (Figure 4B, Table 2), may make significant contributions to the TAK-779 binding pocket, with additional experimentation. However, the very modest effects that we observed from the three experiments suggest that any contribution of these residues to a binding site is minor at most, because in our experience, mutation of key ligand contact points results in a dramatic loss of activity (de Mendonça *et al*., 2005; Vaidehi *et al*., 2006; Wise *et al*., 2007; Hall *et al*., 2009; Nedjai *et al*., 2011). Moreover, we expected TAK-779 to be more sensitive to the effects of mutation within its binding site, as its potency is two orders of magnitude lower than that of VUF 10085 at WT CXCR3 (Figure 1 and Table 1). Based on our previous experiences, we therefore concluded that further assessment of this panel of residues was unlikely to be fruitful in the search for a TAK-779 binding site and was not a good use of resources.

We postulate that the quaternary nitrogen of TAK-779 is critical for the interaction of the compound with CXCR3, because the derived compound TAK-652, which has increased potency and oral bioavailability (Seto *et al*., 2006) is devoid of activity at CXCR3 (data not shown). Possible counterions for the quaternary nitrogen of TAK-779 exist in the TM domains, namely Asp-52^1.31^, Asp-89^2.40^, Asp-112^2.63^, Asp-186^4.60^, Asp-278^6.58^, Asp-282^6.62^, Glu-293^7.28^ and Asp-297^7.32^. Similarly, two negatively charged side chains exist within ECL2, Asp-195 and Glu-196, which we have previously postulated from a salt bridge with residues Lys-125 and Arg-288 in ECL1 and ECL3, respectively, which perhaps TAK-779 disrupts to antagonize CXCR3 function. However, direct examination of point mutants of Asp-112^2.63^, Asp-195, Glu-196, Asp-272, Asp-278^6.58^, Glu-293^7.28^ and Asp-297^7.32^ excluded these residues from interacting with TAK-779.

In summary, we describe the intrahelical binding site of a highly potent, specific CXCR3 antagonist. Knowledge of this binding site may pave the way for the rational design of novel antagonists of this clinically important chemokine receptor.

## Abbreviations

ECL: extracellular loop
HA: haemagglutinin, TM transmembrane
WT: wild type
